# Steviol Glycosides Modulate Glucose Transport in Different Cell Types

**DOI:** 10.1155/2013/348169

**Published:** 2013-11-12

**Authors:** Benedetta Rizzo, Laura Zambonin, Cristina Angeloni, Emanuela Leoncini, Francesco Vieceli Dalla Sega, Cecilia Prata, Diana Fiorentini, Silvana Hrelia

**Affiliations:** ^1^Department for Life Quality Studies, Alma Mater Studiorum, University of Bologna, Corso Augusto 237, 47921 Rimini, Italy; ^2^Department of Pharmacy and Biotechnology, Alma Mater Studiorum, University of Bologna, Via Irnerio 48, 40126 Bologna, Italy

## Abstract

Extracts from *Stevia rebaudiana* Bertoni, a plant native to Central and South America, have been used as a sweetener since ancient times. Currently, *Stevia* extracts are largely used as a noncaloric high-potency biosweetener alternative to sugar, due to the growing incidence of type 2 diabetes mellitus, obesity, and metabolic disorders worldwide. Despite the large number of studies on *Stevia* and steviol glycosides *in vivo*, little is reported concerning the cellular and molecular mechanisms underpinning the beneficial effects on human health. The effect of four commercial *Stevia* extracts on glucose transport activity was evaluated in HL-60 human leukaemia and in SH-SY5Y human neuroblastoma cells. The extracts were able to enhance glucose uptake in both cellular lines, as efficiently as insulin. Our data suggest that steviol glycosides could act by modulating GLUT translocation through the PI3K/Akt pathway since treatments with both insulin and *Stevia* extracts increased the phosphorylation of PI3K and Akt. Furthermore, *Stevia* extracts were able to revert the effect of the reduction of glucose uptake caused by methylglyoxal, an inhibitor of the insulin receptor/PI3K/Akt pathway. These results corroborate the hypothesis that *Stevia* extracts could mimic insulin effects modulating PI3K/Akt pathway.

## 1. Introduction

*Stevia rebaudiana* Bertoni is a weak perennial shrub belonging to Asteraceae (Compositae) family, native to subtropical regions of Brazil and Paraguay. Its leaves have been used as a sweetener since ancient times and for many other medicinal purposes in Latin America and the Orient for centuries [1, 2]. The “sweet herb” has gained increasing interest from nutritional researchers and commercial area in the last years, due to the growing need to find new natural calorie-free sweeteners alternative to sugar. Indeed, in both industrialized and developing countries, the incidence of type 2 diabetes mellitus and obesity is sharply increasing as a result of dietary behaviours, reduced physical activities, and ageing. These metabolic disorders have become major public health problems worldwide [3, 4].

Glycemic control is fundamental to the management of diabetes since it is associated with significantly decreased rates of retinopathy, nephropathy, neuropathy, and cardiovascular disease, the most common cause of death in diabetic patients. The effort to achieve near-normoglycemia through the key strategy of glycemic control includes recommendations for prevention and control of diabetes, for example, monitoring carbohydrate intake and limiting the consumption of sugar-sweetened beverages [5].

**Figure 1:**
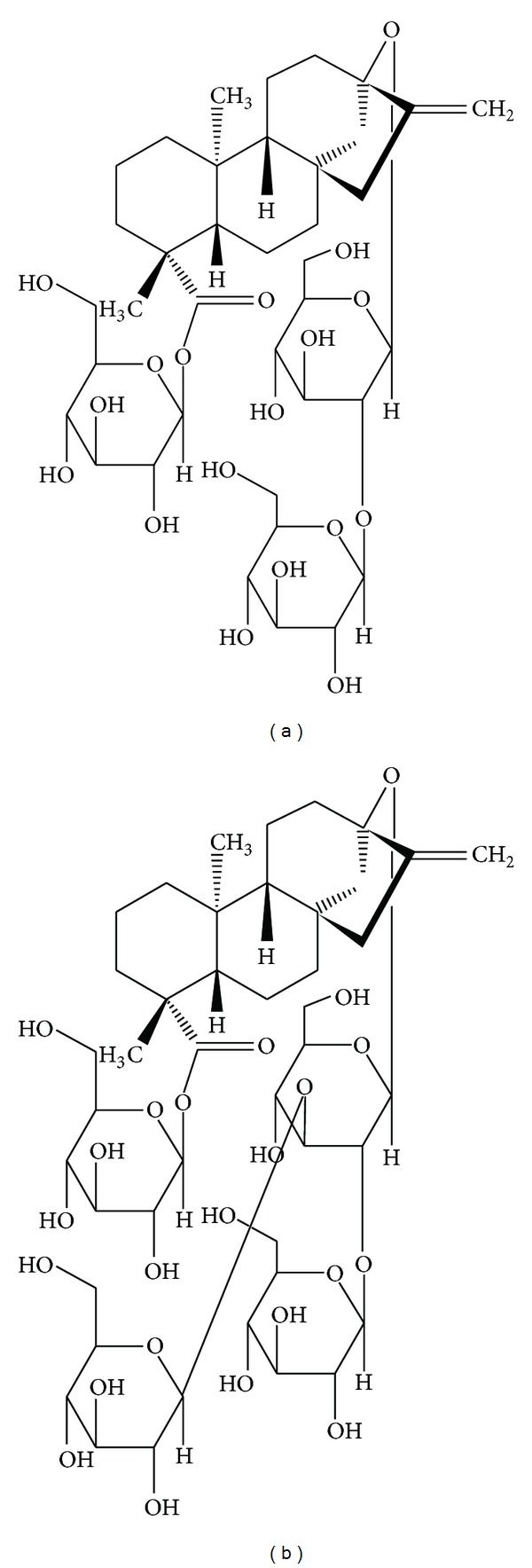
Chemical structure of stevioside (a) and rebaudioside A (b).

*Stevia* leaves and extracts are natural noncaloric sweeteners that can substitute sucrose. The main sweet components in leaves, approximately 200–400 times sweeter than sucrose as shown by organoleptic tests [1, 6], are stevioside and rebaudioside A, steviol glycosides differing only by one glucose moiety. Stevioside is formed by 3 molecules of glucose and one molecule of the aglycone steviol, a diterpenic carboxylic alcohol; rebaudioside A holds one additional glucose molecule [7] (Figure 1).

Steviol glycosides have been recently authorised as commercial sweeteners. US Food and Drug Administration (FDA) has allowed the use of *Stevia* extracts containing not less than 95% total steviol glycosides. Recently, the European Food Safety Authority (EFSA) approved the use of steviol glycosides as food additive [8, 9]. Considering the available toxicity data (*in vitro* and *in vivo* animal studies and some human tolerance studies), steviol glycosides are considered not carcinogenic, genotoxic, or associated with any reproductive/developmental toxicity. Joint Expert Committee on Food Additives (JECFA) established an accepted daily intake (ADI) for steviol glycosides (expressed as steviol equivalents) of 4 mg/kg bw/day [10, 11].

Besides sweetness, steviol glycosides, in particular stevioside, have been shown to possess beneficial effects on human health [7, 12, 13]. Briefly, pharmacological activities and therapeutical benefits include antitumour and anticancer, antiinflammatory, antihyperglycemic, antihypertensive, antidiarrheal, immunomodulatory, diuretic, and enzyme inhibitory actions. *Stevia* has also been used to help control weight in obese subjects [14]; moreover, antioxidant properties have been described [15, 16]. Stevioside, rebaudioside A, and their metabolite steviol have been mostly investigated in *in vivo* animal studies and, at a lesser extent, in humans. Results suggest that stevioside and related compounds affect plasma glucose modulating insulin secretion and sensitivity, which increase glucose removal from the plasma [17, 18]. In addition, it seems likely that stevioside inhibits gluconeogenesis in the liver of diabetic rats [19, 20]. These antihyperglycemic, insulinotropic, and glucagonostatic effects, especially for rebaudioside A, are largely plasma glucose level dependent, requiring high glucose levels [21, 22]. Despite the large number of studies on *Stevia* and steviol glycosides, very little is reported concerning the cellular and molecular mechanisms underpinning these effects.

In the present study, we examined the role of steviol glycosides on cellular glucose transport in cultured cells. Glucose is a polar molecule and requires specific carrier proteins, located in the plasma membrane, to cross the lipid bilayer and enter the cell. Glucose is transported into the cells through two different types of membrane associated carrier proteins, the Na^+^-coupled glucose transporters (SGLT) and the facilitative glucose transporters (GLUT). The human GLUT family is integral membrane proteins widely distributed in probably all mammalian cells that regulate the movement of glucose between extracellular and intracellular compartments maintaining a constant supply of glucose available for metabolism [23]. To date, GLUT family is constituted by 14 distinct isoforms, differently distributed in human tissues [23, 24]. GLUT1 is considered responsible for the basal uptake in many cell types, representing the most ubiquitously expressed isoform; GLUT4 is responsible for insulin-stimulated glucose uptake in peripheral tissues, but its expression has also been reported in the brain [25, 26], where glucose is an essential substrate for cerebral oxidative metabolism. It has recently been reported that in a human neuronal cell line, SH-SY5Y, GLUT1 translocation in response to insulin-like growth factor (IGF-I) occurs [27] and, for the first time in a neuronal cell system, also GLUT4 is translocated to the plasma membrane in response to insulin [28]. We have been studying for a long time the glucose transport activity in many leukaemia cell lines expressing mainly GLUT1, demonstrating that, also in these cell types, GLUT1 is recruited on the plasma membrane from intracellular compartments in response to different stimuli, greatly enhancing the rate of glucose uptake [29, 30]. Moreover, it is well known that impaired GLUT4 translocation is causally linked to insulin resistance and consequently to noninsulin-dependent diabetes mellitus [31, 32].

Starting from this knowledge and this background, we chose the neuroblastoma SH-SY5Y and the promyelocytic leukaemia line HL-60, both expressing insulin and insulin-like growth factor-1 (IGF-I) receptors [28, 33] to test some commercial *Stevia* extracts, in order to evaluate a possible effect of these compounds on glucose transport and to clarify the molecular mechanism of action. 

## 2. Materials and Methods

### 2.1. Chemicals and Reagents

Dulbecco's modified Eagle's medium (DMEM), foetal calf serum (FCS), penicillin/streptomycin, 3-(4,  5-dimethylthiazol-2-yl)-2, 5-diphenyltetrazolium bromide (MTT), staurosporine, 2′,7′-dichlorodihydrofluorescein diacetate (H_2_DCFDA, dichlorofluorescin diacetate), 2-deoxy-glucose (DOG), phloretin, CelLytic M, mammalian protease inhibitor mixture, primary antibody to *β*-actin, methylglyoxal (MG), hydrogen peroxide, bovine serum albumin (BSA), rebaudioside A standard (StReb), stevioside standard (StStev), and all other chemicals of the highest analytical grade were purchased from Sigma-Aldrich. Roswell Park Memorial Institute (RPMI) 1640 medium (with Hepes, with L-glutamine) was purchased from PAA. 2-Deoxy-D-[2, 3]-glucose and Ultima Gold MV scintillation cocktail were from PerkinElmer. PhosSTOP, a phosphatase inhibitor cocktail, was obtained from Roche Diagnostic. Nitrocellulose membranes and Amersham ECL Advance Western Blotting Detection Reagents were from GE-Healthcare. Primary antibodies against phospho-Akt (Ser473) (no. 4058), total Akt (no. 9272), and horseradish peroxidase-conjugated secondary antibodies anti-rabbit (no. 7074) and anti-mouse (no. 7076) were purchased from Cell Signaling Technologies. Anti-GLUT1 (sc-1603), anti-GLUT4 (sc-1606) antibodies, and anti-goat IgG conjugated to horseradish peroxidase (sc-2020) were obtained from Santa Cruz Biotechnology. Primary antibody anti-phospho-PI3 Kinase p85 pTyr458/p55 pTyr199 (no. PA5-17387) was from Thermo Scientific. Anti-PI3 Kinase (no. 06-195) antibody was purchased from Millipore. PageRuler Prestained protein ladder was from Fermentas—Thermo Fisher Scientific.

Extracts from *Stevia rebaudiana* Bertoni were kindly supplied by Eridania Sadam SpA.

According to FDA and EFSA [8–11], total content of steviol glycosides in commercial *Stevia* extracts has to be at least 95% (w/w), and rebaudioside A plus stevioside must be at least 75%. The four extracts tested differ by the relative content of rebaudioside A and stevioside. In particular, according to the certificates of analysis of each sweetener, Reb A (R97) contains 97-98% rebaudioside A, *Stevia* RA60 (R60) contains about 60% rebaudioside A and about 20% stevioside; Steviol Glycosides SG95 (SG) contains 50% rebaudioside A and at least 25% stevioside; Truvia (TRU) contains a mixture of steviol glycosides not analytically quantified. 

### 2.2. Cell Culture

SH-SY5Y, human neuroblastoma cells, were grown at 37°C in a humidified incubator with 5% CO_2_ in Dulbecco's modified Eagle's medium (DMEM) supplemented with 10% (v/v) foetal bovine serum (FBS), 2 mM glutamine, 50 U/mL penicillin, and 50 *μ*g/mL streptomycin, as reported in [34]. HL-60, acute myeloid leukaemia cells, were cultured in RPMI-1640 medium supplemented with 10% FBS, 2 mM glutamine, 100 U/mL penicillin, and 100 *μ*g/mL streptomycin, at 37°C in a humidified atmosphere maintained at 5% CO_2_.

### 2.3. Cell Viability

Cells were treated with different concentrations of steviol glycosides (0.5 to 5 mg/mL) or 1 mM (corresponding to 1 mg/mL) StReb or 1 mM (corresponding to 0.8 mg/mL) StStev for 24 h. Cell viability was evaluated by the MTT assay as reported in [35]. SH-SY5Y and HL-60 cells were incubated with 0.5 mg/mL MTT for 4 h at 37°C in multiwell plates. At the end of the incubation, blue-violet formazan salt crystals were formed and dissolved by adding the solubilisation solution (10% SDS, 0.01 M HCl); then the plates were incubated overnight in humidified atmosphere (37°C, 5% CO_2_) to ensure complete lysis. The absorbance at 570 nm was measured using a multiwell plate reader (Wallac Victor^2^, PerkinElmer).

### 2.4. Lactate Dehydrogenase Assay

SH-SY5Y and HL-60 cells were incubated with 1 mg/mL of each *Stevia* extract for 24 h. Lactate dehydrogenase (LDH) release from cells was monitored by collecting aliquots of medium. LDH activity was assayed by a spectrophotometric method based on the reduction of pyruvate to lactic acid coupled to NADH oxidation. The decrease in absorbance at 340 nm was monitored at 37°C. 100 *μ*M H_2_O_2_ for 30 minutes was used as a positive control.

### 2.5. Assay for Caspase 3 Activity

Caspase 3 activity in the cell lysates was measured using a colorimetric assay kit by following the instructions from the manufacturer (Sigma), as described in [36]. Cells were incubated with or without steviol glycosides (1 mg/mL) for 1, 6, or 24 h. After 24 h, cells were collected and lysed using the lysis buffer provided in the kit (250 mM HEPES, pH 7.4 containing 25 mM CHAPS, and 25 mM DTT). The assay was based on the hydrolysis of the peptide substrate acetyl-Asp-Glu-Val-Asp-aminomethylcoumarin (Ac-DEVD-AMC) by caspase 3, resulting in the release of free AMC moiety. The fluorescence of AMC was read using a multiwell plate reader (Wallac Victor^2^, PerkinElmer); excitation and emission wavelengths were 360 nm and 460 nm, respectively.

The concentration of the AMC released was calculated using an AMC standard curve. Caspase 3 activity was expressed in nmole of AMC released per min per mL of cell lysate and normalised for total protein content in the lysate. Results are reported as percentage with respect to the control. Staurosporine (1 *μ*g/mL) was used as an apoptosis inducer (positive control).

### 2.6. Measurement of Intracellular Reactive Oxygen Species (ROS) Levels

ROS intracellular level was evaluated by using the fluorescent probe 2′,7′-dichlorodihydrofluorescein diacetate (H_2_DCFDA). SH-SY5Y and HL-60 cells were incubated with 5 mg/mL of each *Stevia* extract for 1 h and then subjected or not to oxidative stress generated by 100 *μ*M H_2_O_2_ for 30 minutes. Successively, cells were washed twice in PBS and incubated with 5 *μ*M H_2_DCFDA for 20 min at 37°C. H_2_DCFDA is a small nonpolar, nonfluorescent molecule that diffuses into the cells, where it is enzymatically deacetylated by intracellular esterases to a polar nonfluorescent compound, that is oxidised to the highly green fluorescent 2′,7′-dichlorofluorescein (DCF). The fluorescence of oxidized probe was measured using a multiwell plate reader (Wallac Victor^2^, PerkinElmer). Excitation wavelength was 485 nm and emission wavelength was 535 nm. Fluorescence values were reported as the percentage of intracellular ROS with respect to control.

### 2.7. Glucose Transport Assay

Glucose transport assay was performed as described in [37, 38]. Cells were incubated or not with different compounds (1 mg/mL) for 1 h; then they were washed twice in PBS and treated for 10 min (SH-SY5Y) or 2 min (HL-60) at 37°C with a mixture of 2-deoxy-D-[2, 3] glucose (0.8 *μ*Ci/assay) and 1.0 mM unlabeled glucose analogue, under conditions where the uptake was linear at least for 20 min. The transport was stopped by adding phloretin (final concentration 0.3 mM), a potent inhibitor of glucose transport activity. Radioactivity was measured by liquid scintillation counting (Tri-Carb liquid scintillation analyser, PerkinElmer).

### 2.8. Immunoblotting Analysis

After treatments, cells were washed with ice-cold PBS and lysed on ice using CelLytic M containing mammalian protease and phosphatase inhibitor mixture. The resulting lysed cells were left on ice to solubilize for 45 min. The lysates were centrifuged at 5000 g for 5 min at 4°C to remove unbroken cell debris and nuclei. Cell lysate protein concentration was determined by the Bio-Rad Bradford protein assay (Bio-Rad Laboratories). Samples were kept at 95°C for 5 min prior to separation on 10% SDS-PAGE Mini-Protean TGX precast gels using a Mini-Protean apparatus (Bio-Rad Laboratories). Proteins (15 *μ*g/lane) were electrophoretically transferred to nitrocellulose membrane (Hybond-C; GE Healthcare) in Tris-glycine buffer at 110 V for 90 min. Membranes were then incubated in blocking buffer containing 5% (w/v) albumin in Tris-buffered saline (TBS)/Tween to avoid nonspecific binding and incubated overnight at 4°C with primary antibodies (anti-GLUT1, anti-GLUT4, anti-phospho-Akt, anti-total Akt, anti-phospho-PI3K, anti-total-PI3K, or anti-*β*-actin as internal normalizer). Nitrocellulose membranes were then washed 3 times with TBS/Tween and incubated with secondary antibodies in TBS/Tween containing 5% albumin for 60 min at room temperature and successively washed with TBS/Tween. The results were visualized by chemiluminescence using ECL Advance reagent according to the manufacturer's protocol (GE Healthcare). Images of the blots were obtained using a CCD imager (ChemiDoc MP System, Bio-Rad). Bands were acquired and analysed by using Image Lab analysis software.

### 2.9. Statistical Analysis

Results are expressed as means ± SD. Differences among the means were determined by Bonferroni multiple comparison test following one-way ANOVA and were considered significant at *P* < 0.05.

## 3. Results and Discussion

The effect of different commercial extracts from *Stevia rebaudiana* Bertoni on glucose transport was investigated in both SH-SY5Y neuroblastoma and HL-60 myeloid leukaemia human cells.

Glucose is the primary source of energy used by the brain and it is constantly delivered to individual cells (glial cells and neurons) [39]. In brain, the relationship among glucose metabolism, GLUT isoforms, modulation of glucose uptake, role of insulin, and distribution of insulin receptor (IR) is very complex, being dependent on specific regions of the brain and playing a key role also in cognitive functions. Recent studies report a close correlation between impaired glucose uptake/metabolism and neurodegenerative diseases such as Alzheimer's disease [40–42].

It is also recognised that cancer cells frequently overexpress the GLUT family members, due to the uncontrolled proliferation requiring elevated energy, and they often express GLUT isoforms not present in normal conditions. Moreover, large hypoxic areas into the tumour cause an increase in glucose utilization by cancer cells through glycolysis. The requirement for energy is satisfied by an augmented sugar intake, realised by an increase in GLUT expression and an increment in the translocation of the transporters to the plasma membrane [23]. For these reasons, cancer cells are a useful model system to study the glucose transport activity and its signalling transduction pathway, allowing to clarify the molecular mechanism underlying steviosides biological effects on glucose metabolism.

**Figure 2:**
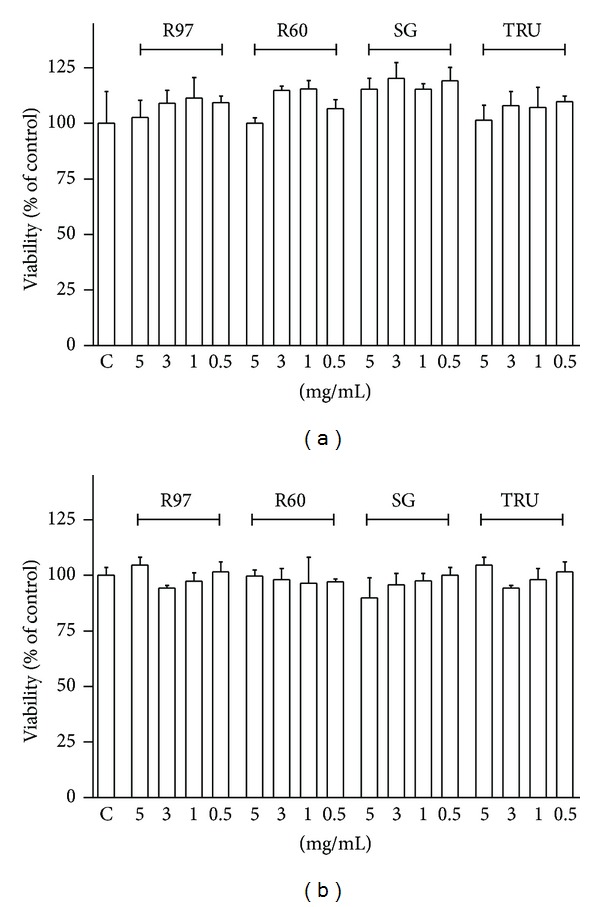
Effect of steviol glycosides on cell viability/proliferation. SH-SY5Y (a) and HL-60 (b) cells were treated for 24 hours with different concentrations of the four compounds (0.1 mg/mL to 5 mg/mL). Viability/proliferation was evaluated by MTT assay as described in Section 2 and compared to control (C). Results are expressed as means ± SD of three independent experiments (*n* = 8). Statistical analysis was performed by Bonferroni multiple comparison test following one-way ANOVA. Significant differences were not revealed.

The first aim of our paper was to evaluate the effect of four different *Stevia* extracts on cellular viability, assessed by MTT assay. SH-SY5Y and HL-60 cells were treated with different concentrations (0.5–5 mg/mL, corresponding to 0.5–5 mM for R97, which can be assumed as a pure compound) of *Stevia* extracts for 24 h. Data reported in Figure 2 show that the extracts did not affect cell viability/proliferation, confirming that they are not cytotoxic within the concentration range tested. Same results were obtained with similar concentrations of StReb or StStev (data not shown).

**Figure 3:**
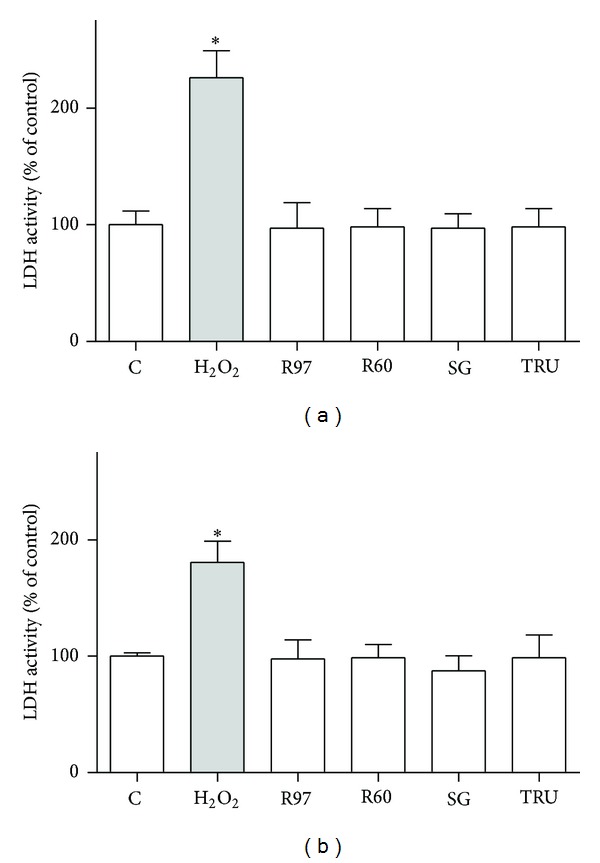
Effect of steviol glycosides treatment on lactate dehydrogenase (LDH) activity.**   **LDH activity was measured by LDH assay as described in Section 2. SH-SY5Y (a) and HL-60 (b) cells were treated with different compounds at 1 mg/mL final concentration for 24 hours, or cells were treated with 100 *μ*M H_2_O_2_ for 30 min as control of LDH activity. Results are expressed as means ± SD of three independent experiments, each performed in triplicate. Statistical analysis was performed by Bonferroni multiple comparison test following one-way ANOVA. *P < 0.05, significantly different from control cells.

Cytotoxicity was also evaluated by lactate dehydrogenase (LDH) assay, which indicated that cell membrane integrity was not compromised and excluded cellular necrosis (Figure 3).

**Figure 4:**
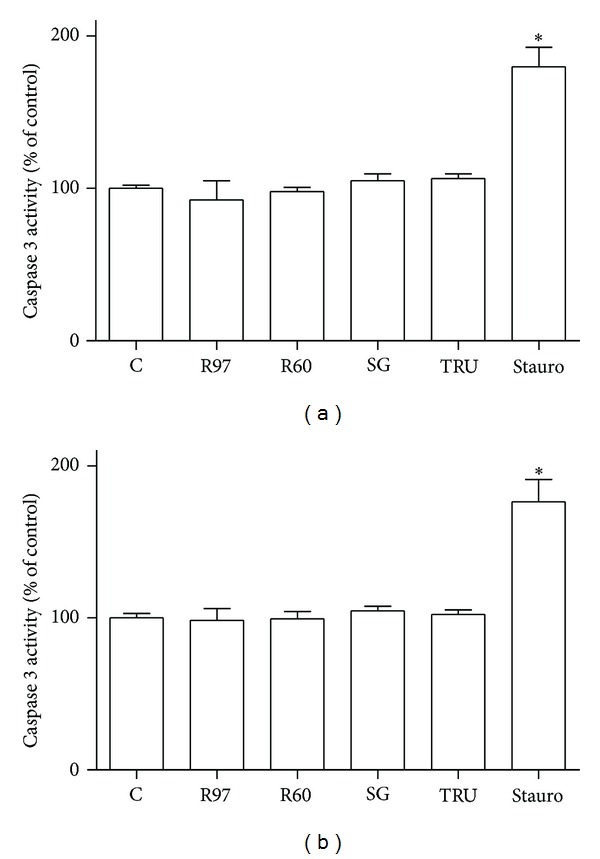
Caspase 3 activity in SH-SY5Y (a) and HL-60 (b) cells was evaluated in the presence of 1 mg/mL steviol glycosides incubated for 24 hours. Staurosporine (Stauro, 1 *μ*g/mL for 4 hours) was used as positive control. Caspase 3 activity was measured spectrofluorimetrically after 24 hours in cell lysates as reported in Section 2. Each column represents the mean ± SD of three independent experiments. Statistical analysis was performed by Bonferroni multiple comparison test following one-way ANOVA. *P < 0.05, significantly different from control cells.

In order to evaluate a possible effect on apoptosis, the activity of caspase 3 was measured. Caspases play a central role in mediating various apoptotic responses and are activated in a sequential cascade of cleavages. To detect the enzymatic activity of caspase 3, the fluorogenic substrate Ac-DEVD-AMC was employed. Treatments of cells with *Stevia* extracts for 1 hour, 6 hours (data not shown), or for 24 hours (Figure 4) did not influence caspase 3 activity, indicating that the compounds did not induce programmed cell death.

**Figure 5:**
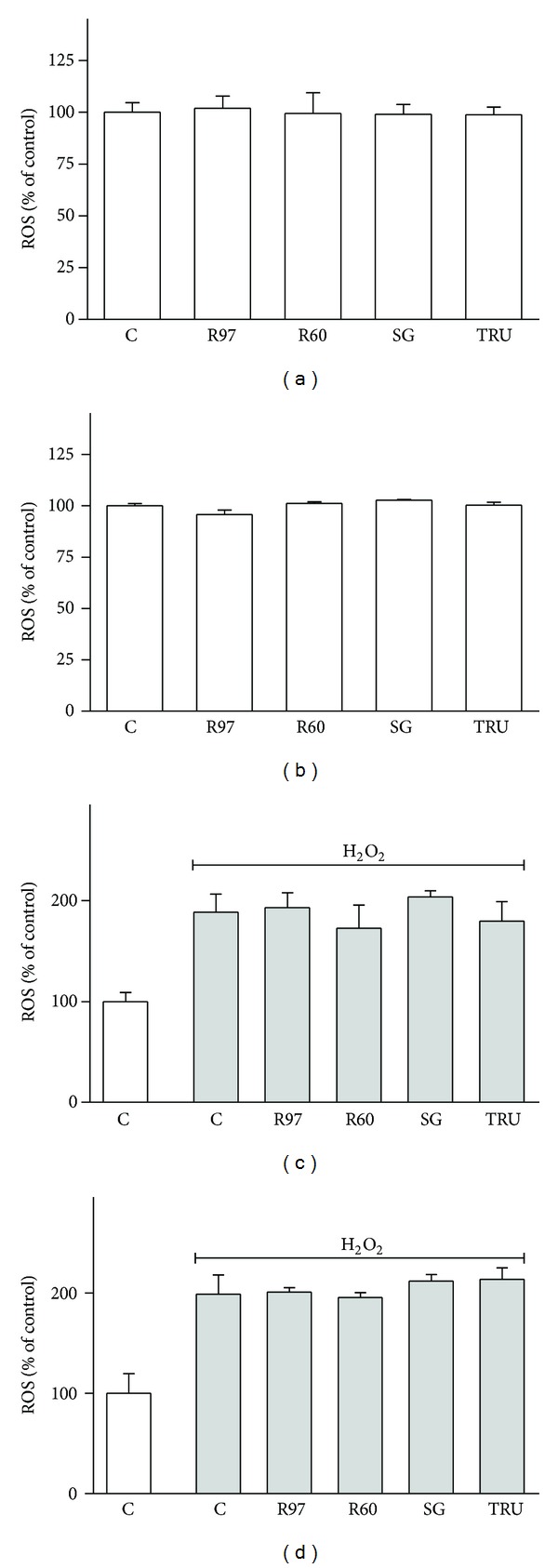
Effect of steviol glycosides on ROS levels in SH-SY5Y and HL-60 cells. SH-SY5Y (a) and HL-60 (b) cells were treated for 1 hour with different compounds (5 mg/mL); then basal ROS levels were measured by means of H_2_DCFDA assay as described in Section 2. Results are expressed as means ± SD of four independent experiments (*n* = 8). Statistical analysis was performed by Bonferroni multiple comparison test following one-way ANOVA. Significant differences were not revealed. SH-SY5Y (c) and HL-60 (d) cells were preincubated for 1 hour with different compounds (5 mg/mL) and then exposed to oxidative stress generated by 100 *μ*M H_2_O_2_ for 30 min. ROS levels were measured by means of H_2_DCFDA assay as described in Section 2. Results are expressed as means ± SD of four independent experiments (*n* = 8). Statistical analysis was performed by Bonferroni multiple comparison test following one-way ANOVA. Significant differences were not revealed among H_2_O_2_ treated cells.

Since antioxidant properties of *Stevia* extracts have been described [15, 16], the antioxidant activity of the commercial extracts was investigated in SH-SY5Y and HL-60 cells. Reactive oxygen species (ROS) levels were measured with the cell-permeant probe H_2_DCFDA, commonly used to detect free radical/ROS production in cells, owing to the intracellular conversion to the highly green fluorescent DCF [43]. As shown in Figure 5, the compounds did not exhibit any antioxidant activity, since they were neither able to decrease basal ROS level at the highest concentration used nor to counteract intracellular ROS raise due to exogenous oxidative stress (100 *μ*M H_2_O_2_ for 30 min). This lack of antioxidant activity is in contrast with the data reported by other authors [15, 16], probably because the compounds used in the present study are commercial sweeteners containing 95–98% steviol glycosides with no appraisable amounts of polyphenols, naturally present in *Stevia* leaves, likely responsible for *Stevia* antioxidant activity.

**Figure 6:**
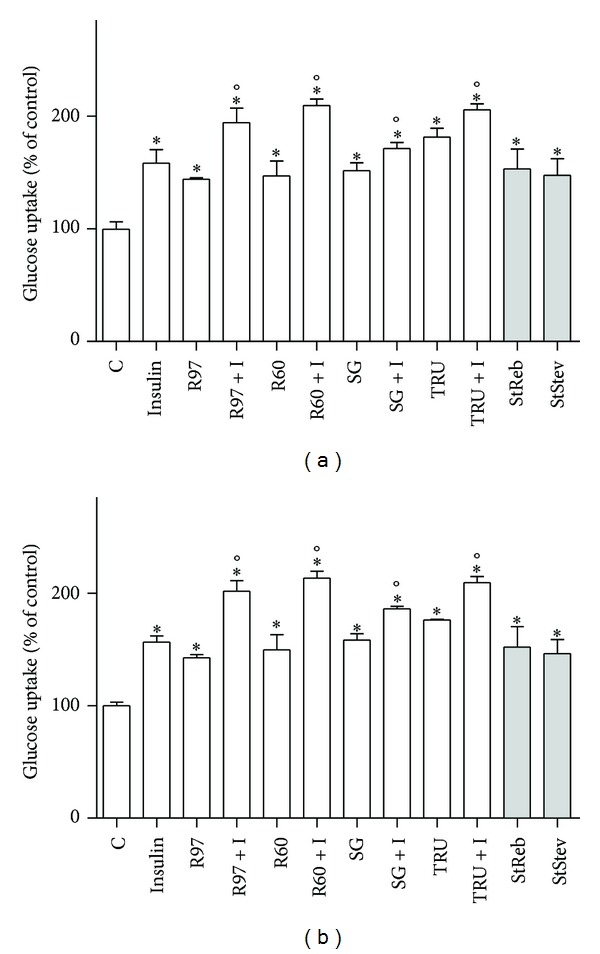
Effects of *Stevia* extracts on glucose transport activity compared to the effect of insulin. SH-SY5Y (a) and HL-60 (b) cells were treated with steviol glycosides (1 mg/mL), with 100 nM insulin (I), with steviol glycosides and insulin simultaneously, or 1 mM standard compounds (StReb, StStev). Glucose uptake was assayed as described in Section 2. Results are expressed as means ± SD of three independent experiments, each performed in triplicate. Statistical analysis was performed by Bonferroni multiple comparison test following one-way ANOVA. *P < 0.05, significantly different from control cells; °*P* < 0.05 significantly different from the corresponding cells not stimulated with insulin.

*Stevia* extracts are largely used as a noncaloric high-potency biosweetener substitute for sugar. The effect of four *Stevia* extracts on glucose transport activity was evaluated in HL-60 human leukaemia cells, expressing principally GLUT1, the basal glucose transporter, and in SH-SY5Y human neuroblastoma cells, expressing also GLUT4, the insulin-sensitive one.  Figure 6 shows that all the extracts and the two standard compounds were able to enhance glucose uptake at similar extent after 1-hour incubation in both cellular lines.

Since the increase in glucose uptake obtained with standards is consistent with that shown by the whole extracts, we can hypothesize that this effect is due to rebaudioside A and stevioside, the two major components of *Stevia* extracts.

Furthermore, the influence of *Stevia* extracts on glucose transport activity was compared to the effect of 100 nM insulin (Figure 6). Results reveal that *Stevia* extracts and insulin behave similarly, being *Stevia* extracts as efficient as insulin in increasing glucose uptake. The cotreatment with insulin and *Stevia* extracts causes a rise of glucose transport significantly higher than the increase due to insulin alone.

**Figure 7:**
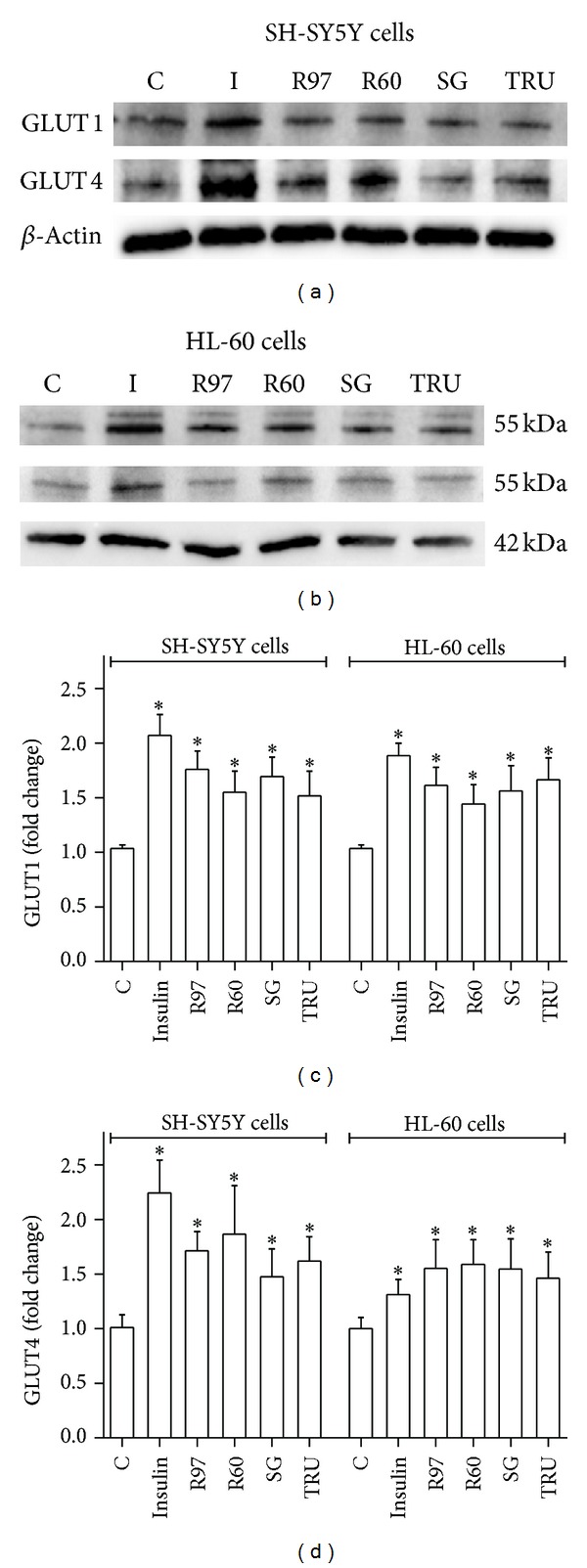
Effects of *Stevia* extracts on GLUT content. SH-SY5Y and HL-60 cells, treated with insulin or with steviol glycosides, were lysed with CelLytic M as described in Section 2. Cell lysates were electrophoresed and immunoblotted with the indicated antibodies, as described in Section 2. *β*-Actin detection was used as a control. Immunoblots representative of three independent experiments are reported for SH-SY5Y (a) and HL-60 (b) cell lines; densitometric analysis (normalized for *β*-actin content and expressed as fold of control) is shown for GLUT1 (c) and GLUT4 (d). *P < 0.05, significantly different from control cells.

It is well known that insulin induces the translocation of GLUT4 from cytosolic storage vesicles to the plasma membrane, enhancing glucose transport. Recently, this phenomenon was observed also for neural cells *in vitro*, in particular the human neuroblastoma SH-SY5Y cells, through a phosphatidylinositol 3-kinase (PI3K)–dependent mechanism similar to the mechanism described in muscle or adipose tissues [28]. Translocation of GLUTs to the membrane has been reported as a consequence of various stimuli in many cellular types; in addition, changes in the expression of GLUTs have been described in response to several metabolic and oxidative stresses and in various physiological or pathological conditions. For example, insulin and ischemia induce GLUT1 movement to the membrane in rat heart [44]; doxorubicin recruits GLUT1 to the plasma membrane by a ROS-mediated mechanism in cardiomyocytes [37]; L-cysteine increases glucose uptake and GLUT3 levels in SH-SY5Y cells [45]; growth factors, cytokines, hydrogen peroxide, and cholesterol depletion are able to increase glucose uptake and GLUT1 translocation in leukaemia cell lines [46–49]. The expression of GLUT1 and GLUT4 in neuroblastoma and leukaemia cells following treatments with *Stevia* extracts or insulin was assessed by Western blot analysis on cell lysates.  Figure 7 reports representative immunoblots for GLUT isoforms and the densitometric analysis in both cell lines. It can be seen that the increase in GLUT1 and GLUT4 content obtained following exposure to *Stevia* extracts is similar to that obtained by insulin stimulation. These results are in accordance with those observed in the evaluation of glucose transport activity.

**Figure 8:**
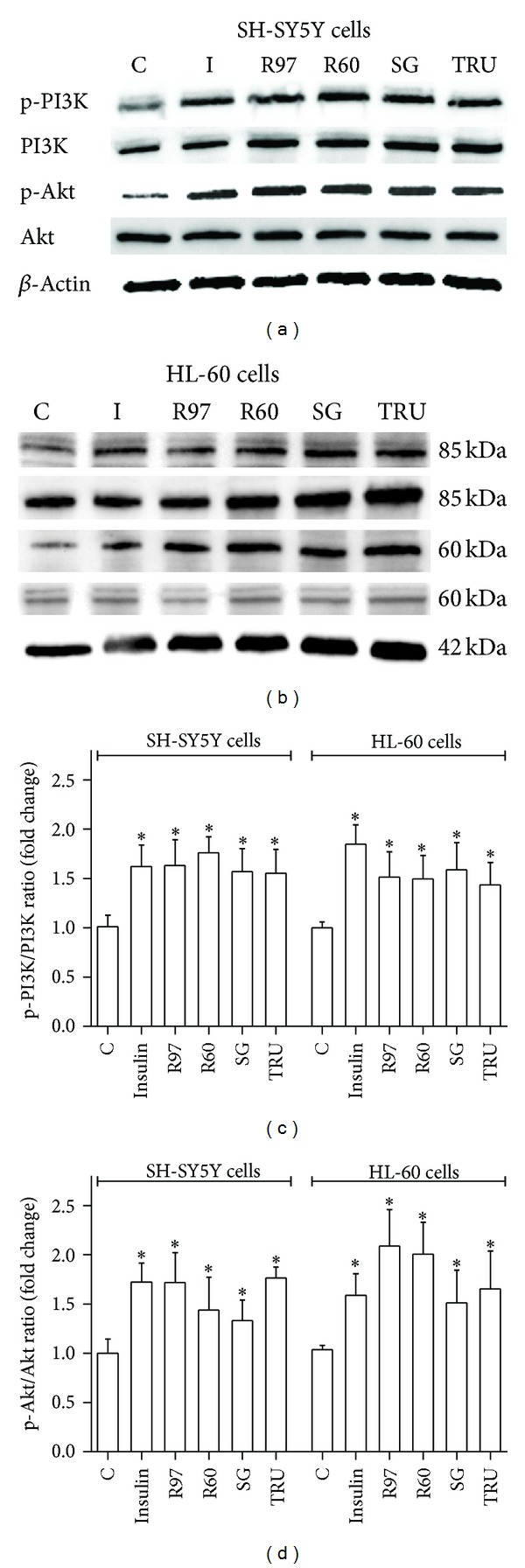
Effects of *Stevia* extracts on PI3K/Akt pathway. SH-SY5Y and HL-60 cells, treated with insulin or with steviol glycosides, were lysed with CelLytic M as described in Section 2. Cell lysates were electrophoresed and immunoblotted with the indicated antibodies, as described in Section 2. *β*-Actin detection was used as a control. Immunoblots representative of three independent experiments are reported for SH-SY5Y (a) and HL-60 (b) cell lines; densitometric analysis of PI3K phosphorylation status is expressed as phospho-PI3K/total PI3K ratio (c) and densitometric analysis of Akt phosphorylation status is expressed as phospho-Akt/total Akt ratio (d). *P < 0.05, significantly different from control cells.

To clarify the molecular mechanism by which *Stevia* extracts enhance glucose transport, the phosphorylation status of PI3K and Akt was evaluated following *Stevia* extract treatment and insulin stimulation. Insulin activates the PI3K/Akt pathway, critical for neuronal survival and growth, synaptic plasticity and development, and learning [50, 51]. Indeed, stimulation of insulin receptor, localized in lipid rafts [52, 53], produces the phosphorylation of tyrosine receptor kinases and the activation of a signal transduction pathway involving PI3K and Akt. The interaction of insulin with its receptor is a regulator of growth and differentiation of leukaemia cells [54, 55]. Highly specific insulin receptors have been identified on human promyelocytic leukaemia cells HL-60 [56]. Immunoblotting results (Figure 8) show an increase of phosphorylated forms of both PI3K and Akt following the treatment with insulin or *Stevia* extracts, indicating a possible similar mechanism of action or, at least, a common signalling pathway.

To better characterize the mechanisms of glucose uptake induction by steviol glycosides, we used methylglyoxal (MG) as an inhibitor of the insulin receptor/PI3K/Akt pathway. MG is a reactive ketoaldehyde, product of many metabolic pathways, primarily glycolysis, which is considered the most relevant and reactive glycation agent *in vivo*. Advanced glycation end products (AGEs) have been implicated in development and progression of several diseases, including diabetes and its associated vascular complications, renal failure, cirrhosis, aging, and recently also in diabetic neuropathy and in Alzheimer's disease [57]. Furthermore, recent studies suggest a correlation between MG and insulin resistance [58].

**Figure 9:**
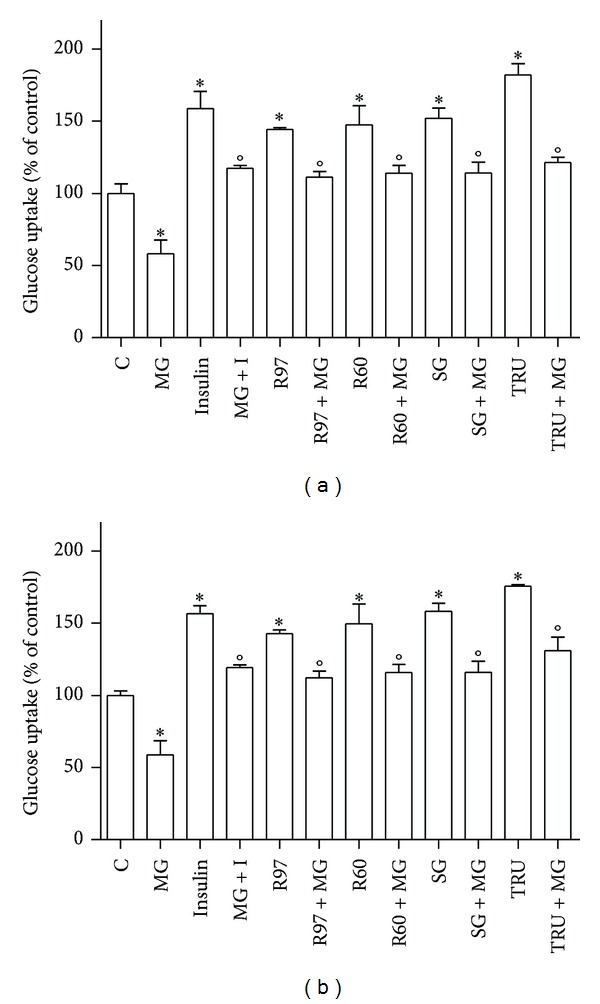
Effects of *Stevia* extracts on glucose transport activity compared to the effect of 0.1 mM of methylglyoxal. SH-SY5Y (a) and HL-60 (b) cells were treated with steviol glycosides, with insulin (I), with methylglyoxal (MG, 2 hours), and with steviol glycosides or insulin in the presence of MG. Glucose uptake was assayed as described in Section 2. Results are expressed as means ± SD of three independent experiments, each performed in triplicate. Statistical analysis was performed by Bonferroni multiple comparison test following one-way ANOVA. *P < 0.05, significantly different from control cells; °*P* < 0.05, significantly different from cells treated with MG alone.

Figure 9 shows that MG incubation (2 hours) induces a significant decrease in glucose transport and that the subsequent treatment with *Stevia* extracts or insulin is able to raise the glucose uptake, corroborating the hypothesis that *Stevia* extracts could act *via* PI3K/Akt pathway similar to insulin. As MG is a reactive aldehyde toxic to cells, MTT assay was performed to verify that the MG concentration (0.1 mM) used to inhibit glucose transport did not influence cell viability (data not shown).

## 4. Conclusions

In this study, we evaluated the effects of steviol glycosides, extracted from *Stevia rebaudiana* Bertoni leaves, on glucose transport activity in two different cell lines. We demonstrated, for the first time to our knowledge, that rebaudioside A and stevioside, the major glycosides in *Stevia* extracts, are able to enhance glucose uptake in both SH-SY5Y neuroblastoma and HL-60 myeloid leukaemia human cells, the raise being similar to that induced by insulin. Our data suggest that steviol glycosides act by modulating GLUT translocation through the PI3K/Akt pathway. Although further experiments are needed, these results support the hypothesis that steviol glycosides and insulin could share a similar mechanism in regulating glucose entry into cells. In conclusion, *Stevia* extracts, commercialised as zero-calorie natural sweeteners, are involved in insulin regulated glucose metabolism. These findings suggest that the use of *Stevia* extracts goes beyond their sweetening power and may also offer therapeutic benefits, supporting the use of botanicals dietary supplements to improve the quality of life.
